# Traditional Practices and Consumer Habits regarding Consumption of Underutilised Vegetables in Kilimanjaro and Morogoro Regions, Tanzania

**DOI:** 10.1155/2020/3529434

**Published:** 2020-08-04

**Authors:** James S. Chacha, Henry S. Laswai

**Affiliations:** Department of Food Technology, Nutrition and Consumer Sciences, College of Agriculture, Sokoine University of Agriculture, P.O. Box 3006, Chuo Kikuu, Morogoro, Tanzania

## Abstract

**Background:**

Wild vegetables serve an important purpose in the health and diet of many people residing in the rural areas. Various traditional practices associated with their processing and consumption are uncommon and unknown to the present generation, resulting to their underutilization.

**Methods:**

Qualitative data were obtained through focus group discussions (FGDs), key informant interviews (KIIs), and participant observation. Using a checklist and questionnaire, a total of 120 individuals from 120 randomly selected households in Kilimanjaro and Morogoro regions were interviewed*. Results*. Underutilised indigenous vegetables from Morogoro and Kilimanjaro regions were investigated. Out of 40 vegetables, four underutilised vegetables (UVs), Sunga (*Launea cornuta*), Kikundembala (*Vigna vexillata*), Mokiki (*Momordica foetida*), and Inyiri (*Basella alba*), were identified, collected, and studied. Over 75% of respondents preferred UVs to exotic species, the likes of cabbage, amaranth, eggplant, pumpkin leaves, and spinach. It was further noted that with the existing myths and fads associated with consumption of UVs, as well as the widespread use of the exotic vegetable species among the modern generation, UVs' future demand is diminishing.

**Conclusion:**

There are many varieties of UVs in Morogoro and Kilimanjaro regions. However, the UVs are currently showing decreasing demand due to various reasons including difficulties in preparation, low palatability, and negative image to consumers; introduction of exotic species of vegetables was found to further suppress the use of UVs.

## 1. Background

Traditional foods are a large and heterogeneous group of raw and processed foods comprising wild indigenous flora and fauna obtained from uncultivated land, forest and aquatic environments and the locally available staple foods processed using traditional processing technologies [1]. Besides the foods being culturally acceptable, they are an integral part of local food habits, which can be collected for consumption or traded locally with no or low commercial value [2]. Wild edible plants (WEPs) have continued to be an important dietary component [3], contributing to food baskets and livelihoods in the smallholder and subsistence farming communities of sub-Saharan Africa [4]. Often referred to as famine or hunger foods, wild plants have been acknowledged to be prospective in meeting household food and income security [5]. These wild ranges of leafy vegetables, roots, tubers, fruits, and stems are harvested by rural communities because of their taste, cultural uses, as food supplements, or to tide over food shortage [6].

Wild vegetables play significant roles in the livelihood of many communities in developing countries as food and for medicinal purposes [7]. They contribute to human welfare significantly as they have huge quantities of vitamins and minerals like folic acid, vitamin A, and vitamin C and potassium, magnesium, calcium, iron, and zinc, respectively [8, 9]. Besides, they have numerous health potentials; for instance, amaranth vegetables are consumed and they are useful in protection against respiratory disorders, defective vision, recurrent colds, retarded growth, and functional sterility [10]. Green leafy vegetables are the powerhouse of health-promoting phytochemicals and can be used by the people of all ages, also they play a key role in alleviation and fighting against many deficiency diseases [11]. The consumption of vegetables has been linked with reduced threats of death from various causes including heart ailments and malignancies, raising an alarm for their increased intake [12]. Therefore, a better understanding of ethno-botanical knowledge is necessary to inform agricultural development, natural resource management, and food security policies that could facilitate more sustainable access to these resources and even increase their positive impact on community resilience [13].

## 2. Methods

### 2.1. Study Areas

The study was conducted in Morogoro and Kilimanjaro regions in Tanzania. These regions were purposely selected as an Eastern and Northern zonal representation of Tanzanian regions participating in agriculture. Two districts were selected from each region: Kilosa district (Morogoro region) lies between latitudes 5°55′ and 7°53′S and longitudes 36°30′ and 37°30′E and within an altitude of 200 to 700 m above the sea level. This district covers a total area of 14,918 km^2^. Mvomero district (Morogoro region) lies between latitudes 05°80′ and 07°40′S and between longitudes 37°20′ and 38°05′E. It covers a total area of 7,325 km^2^. Rombo district (Kilimanjaro region) lies between latitudes 2°50′ and 3°23′S and longitude 37°15′ and 37°41′E and covers an area of 1,442 km^2^. Hai district (Kilimanjaro region) lies between 2°50′S and 3°29′S and longitude 30°30′E and 37°10′E; covering an area of 13,000 km^2^.

Kilosa district has a bimodal rainfall distribution, with short rains starting from October to December while the long rains are between January and May. The highest parts of the district get annual rainfall of 1000-1600 mm whereas the central and southern parts get an average of 800 to 1400 mm. Temperature varies between 15 and 32°C with mean annual temperature of 25°C. The main economic activity carried out in Kilosa district is agriculture (including crop farming and livestock keeping). Mvomero district has temperature range from 18 to 30°C, with annual rainfall from 600 mm to 1000 mm. The area experiences bimodal rainfall pattern where long rains are from March to the end of May, and short rains occur from October to December. The dry seasons are from June to August and January to March. The district economy depends mainly on agriculture.

Rombo district has volcanic soils, with the rainfall pattern being bimodal: short rains from November to December and long rains from March to May. Rainfall ranges from 1000 mm to 2000 mm on average and vary with elevation while temperature ranges from 18 to 28°C. The natives depend on subsistence and small-scale farming and livestock keeping and some depend on retail business. Hai district experiences two main rainy seasons: the long rains which begins in March and ends in June and the short rains that starts in November and ends in December. The area has soils that are mainly alluvial and volcanic in nature and experiences a temperature of 20°C and an average annual rainfall of 700 mm. Most people earn their living through farming, livestock keeping, and trade.

### 2.2. Research Design

Cross-sectional research design was used whereby in a period of one month, data were collected in Morogoro and Kilimanjaro regions. A household survey was carried out to obtain qualitative data on traditional practices and consumer habits regarding the UVs.

### 2.3. Sampling and Sample Size

Two districts from each region were purposively selected based on their extent of participating in agricultural activities, crop farming and livestock keeping, respectively. These were Mvomero and Kilosa districts from Morogoro region and Hai and Rombo districts from Kilimanjaro region. From each district, a single ward was selected using simple random sampling, in which a total of 30 individuals from each ward were selected based on their period of stay in the area, knowledge, experience, and acquaintance with the UVs. Thus, a total of 60 respondents from each region were included in the interviews, making it a total of 120 respondents used in this study. According to Saunders et al. [14], statisticians have shown that a sample size of 30 or more will usually result in a sampling distribution for the mean that is very close to a normal distribution, therefore the sample size used for the study was considered sufficient.

Selection of participants for FGDs was based on the depth of their knowledge on indigenous vegetables, whereby to obtain a detailed set of information, they were divided into separate groups of male and female FGDs. Village leaders assisted in identifying the individuals. Key informants were purposively selected through their various leadership positions in the respective districts. These were village agricultural officers, ward agricultural officers, and the district agricultural officers. Moreover, few notable respondents were also interviewed as key informants based on their long stay (35 years) in the regions as well as being well informed with the UVs due to their old age. Participant observation was also necessary to supplement the information from the respondents, especially to obtain the real situation of the habitats of the UVs and their key features as well as taking photos for identification.

### 2.4. Qualitative Data Collection Methods

Using a checklist and a structured questionnaire, a total of 120 individuals from 120 households in Kilimanjaro and Morogoro regions were interviewed. Data was obtained through focus group discussions (FGDs), key informant interviews (KIIs), and participant observation. The aim of the discussions and interviews was to find out the reasons for consuming the vegetables and whether they had any perceived medicinal/health potentials. The information obtained included names of plant(s) and part(s) used as medicine, forms of administration, habitat, seasonality/availability, processing methods, storage methods, and perceived disease cure as judged by the respondents.

### 2.5. Data Analysis

Data analyzed using Statistical Package for Social Sciences (SPSS) version 16.0. Descriptive statistics such as frequencies and percentages were computed and integrated with summaries from FGDs to draw inferences.

## 3. Results and Discussion

### 3.1. Names of Underutilised Vegetables Screened

From the focus group discussions conducted in Morogoro and Kilimanjaro regions, over 40 UVs were mentioned by the respondents (Table 1).

For the purpose of this study, two vegetables were chosen from each region for laboratory micronutrient analysis, one UV per district. These were *Launea cornuta* and *Vigna vexillata* from Morogoro region and *Momordica foetida* and *Basella alba* from Kilimanjaro region (Table 2). Criteria for selection of the four vegetables were preference of use over the others, availability of the vegetable in the region during the study period, and the belief of having both nutritional and medicinal potentials. In a study by Chacha and Laswai [9], the vegetables were found to be rich sources of vitamins (A, B_1_, B_2_, B_3_, and C) and minerals (Ca, Fe, Mg, and Zn).

#### 3.1.1. Sunga (Bitter Lettuce, *Launea cornuta*)

**Figure 1:**
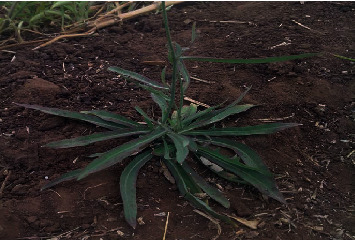
*L. cornuta.* Source: Field survey, 2017.

*(1) Morphological Description*. *L. cornuta* form an arrangement of horizontally spreading leaves at the base, alternate on the stem (Figure 1). The vegetable was reported to be wild, at times, bearing flowers that are usually yellow or whitish in colour, the results being in line with a study by Misonge et al. [15]. It has long and narrow leaves, which contain white fluids and taste very bitter when cooked. It was also reported by the respondents that when properly dried and stored, the leaves can last 6-12 months. *L. cornuta* commonly known as bitter lettuce is a wild vegetable in the family Asteraceae/Compositae [15, 16].

*(2) Acquisition, Processing, and Storage of L. cornuta*. Tender leaves (edible portion) are usually obtained through plucking. Other consumers prefer uprooting the whole plant, which seems like an easy and fast way of acquisition, but a rather troublesome method as one will be required again to detach the leaves from the main stems at home.

*L. cornuta* is usually subjected to various treatments before consuming, including washing, boiling, and drying. The first step involves washing, to remove soil and dirt that might have remained attached to the plant after plucking or uprooting. This is followed by boiling, which can be done up to three times with discarding boiling water each time and replacing it with fresh water, the main reason being to reduce bitterness, a fact that is also supported by Fashir et al. [3]. After several times of boiling and spilling of the water used in boiling, the leaves can be cooked in any form depending on the needs of the consumer. Nevertheless, the bitterness of the vegetable does not completely disappear even after the repeated boiling and spillage of the water, which makes it necessary at times to add spices for suppressing the bitter taste. Bitterness attribute is associated with the milky juice obtained from the leaves [15].

The vegetable is stored in a dried form (sun-dried), which to a small extent results to losses of the leaves' greenish colour which might also imply losses of light-sensitive vitamins like the carotenoids and beneficial pigments like chlorophylls from the vegetable. The dried form of the vegetable, usually kept in a clay pot, can stay in good form for a week. When one needs to use it, the desired amount is taken, rehydrated, and prepared following the normal cooking procedures.

#### 3.1.2. Kikundembala (Wild Cowpea, *Vigna vexillata*)

*(1) Morphological Description*. *Vigna* is a genus of the important legume tribe Phaseoleae, which also contains soybeans and the so-called common bean, *Phaseolus vulgaris* L. [17]. Commonly referred to as the “wild cowpea,” *V*. *vexillata* is native of Africa, Asia, and Central America [18]. It belongs to the family Fabaceae [18, 19].

**Figure 2:**
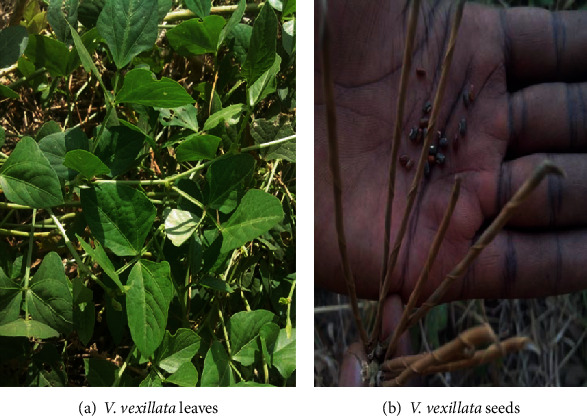
(a) *V. vexillata* leaves. (b) *V. vexillata* seeds. Source: Field survey, 2017.

The morphology of the leaves is not different from that of the legume cowpeas (*Vigna unguiculata*) (Figure 2(a)). The major difference seems to be utilisation and seed edibility, whereby while *V. vexillata* is underutilised as well as wild in nature, *V. unguiculata* is domesticated and highly utilised. In addition to that, the seeds of *V. vexillata* (Figure 2(b)) are inedible, but those of *V. unguiculata* are among the prominent sources of proteins. *V. vexillata* has a tap root system which grows so deep, making it hard to uproot. As supported by Sprent et al. [17], the vegetable was observed to have a special characteristic of spreading and covering a large surface on land.

*(2) Acquisition, Processing, and Storage of V. vexillata*. Usually, the tender new emerging leaves are the ones that are plucked as they are the most suitable for food. This is due to the fact that old leaves are regarded as hard, fibrous, and not easily digestible. Drying and boiling are the major methods of processing the vegetable. The two processes are also associated with storage. Moreover, boiling goes hand in hand with cooking, whereby oil and other spices can be added to increase taste. The vegetable is stored in a dried form, usually preceded by boiling, then kept in a vessel, especially well-dried clay pot. The storability was said to be one year provided that the conditions are favourable.

#### 3.1.3. Mokiki (Bitter Cucumber, *Momordica foetida*)

**Figure 3:**
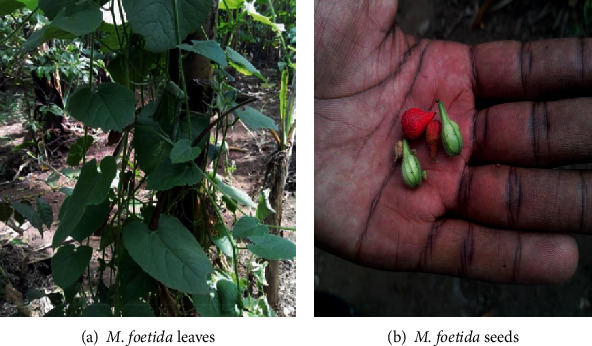
(a) *M. foetida* leaves. (b) *M. foetida* seeds. Source: Field survey, 2017.

*(1) Morphological Description*. *M. foetida* grows by spreading/climbing on other plants, vegetation, or even sticks using its tendrils. Widely known to be a seed-bearing leafy vegetable, *M. foetida* has soft, broad, and spoon-shaped leaves (Figures 3(a) and 3(b)). Its leaves when plucked from the mother plant give a strong and very unpleasant smell (stench), which surprisingly disappears on cooking!

*M*. *foetida* is a medicinal plant that belongs to the family Cucurbitaceae [20]. It is a dioecious, perennial climbing herb with tendrils, rooting at nodes and with dark green, flecked stem and simple succulent leaves. Commonly known as bitter cucumber, plants from the *Mormodica* genus, Curcubitaceae family are consumed as vegetable and are characterized by a bitter taste owing to the presence of phytochemicals [21, 22].

*(2) Acquisition, Processing, and Storage of M. foetida*. Tender leaves are ones that are usually plucked as they are claimed to be suitable and containing the desired components. Two popular processing methods used include cooking/boiling or frying.

A prominent greenish dish referred by the natives to as the “*Moviro*” food can also be prepared using *M. foetida*. The food is prepared through plucking leaves of the vegetable, frying or cooking them, then grinding before squeezing to obtain the greenish liquid from the procedure. The liquid obtained is stirred before mixing with bananas and cooked with milk as one of the ingredients. The whole mixture turns green due to the effect of *M. foetida* inclusion. As gathered from the discussions, the dish is claimed to be very delicious and well known for increasing strength (energy provision) and quick recovery from illness.

Most Rombo district inhabitants do not have a custom of storing vegetables for future use as was the case traditionally, as most of them are used to exotic species. It was also claimed that since the UVs are abundant and naturally growing in farms, there was no need of preserving them.

#### 3.1.4. Inyiri (Malabar Spinach, *Basella alba*)

**Figure 4:**
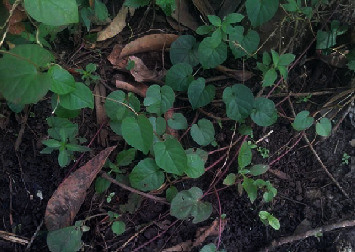
*B. alba*. Source: Field survey, 2017.

*(1) Morphological Description*. Besides being wild in nature, it was learnt from the focus group discussions and participant field observation that *B*. *alba* spreads on ground surface and on other vegetation as a climber plant (Figure 4). Additionally, it was known that the vegetable does not bear seeds, that its stem cuttings are the ones which are planted. When cooked, it behaves slippery like *Corchorus olitorius.*

Numerous studies conducted have shown that *B. alba* is a fast-growing perennial vine which belongs to the family Basellaceae [23]. It is commonly known as Malabar spinach [24, 25]. It has nodes and internodes and can be cultivated from either seed or cutting. It has a bland mucilaginous taste, without odour. They are of three common types: *B*. *alba* with green stem and oval to almost round leaves, *B*. *rubra* with red stems and green and oval to round leaves, and the third type being a hybrid of the two [26]. Their long stems were reported to be used by children as jumping ropes.

*(2) Acquisition, Processing, and Storage of B. alba*. *B*. *alba* leaves are obtained through plucking. As food, *B. alba* leaves are plucked, washed, cut, and then cooked just like other vegetables using tomatoes, onions, or any other condiments. The vegetable can be mixed with bananas, yoghurt, and maize that has been boiled, and the husk has been removed to form a special kind of traditional foodstuff/meal referred by the natives to as “*Kitalolo*”; which is eaten just as is, with no other accompaniment and is claimed to provide a lot of energy and nutrients. According to Mahr [27], young leaves can be eaten raw mixed in green salad and steamed or boiled to be used like cooked spinach, with some people preferring addition of small amounts of vinegar to the cooked leaves to overcome the somewhat slimy texture besides improving palatability.

For medicinal purposes, leaves, stems, and roots of the vegetable are boiled, and the obtained liquid is drank as treatment for constipation and other ailments. The Hai district natives do not store vegetables including *B. alba* at all due to the reason that, just like other UVs, *B. alba* is plenty even though it is not used by many people. Most of the inhabitants are used to buying the exotic vegetables from vendors on daily routine.

### 3.2. Drivers for Interests and Preferences of the UVs

From the FGDs, it was reported that most of the individuals who used the UVs had interest in the vegetables. Most of them were accustomed to UVs since the eras of their great grandparents who were explained to be major consumers of the vegetables on a daily basis, to the extent of staying physically fit, unattacked by the common maladies that befall most people in the present-day generation. They explained that the UVs had a nice taste, besides being, medicinally, a reliable “immunity booster.” Lack of interest and inability to tolerate absurd tastes, for instance the bitterness in *L*. *cornuta* and the foul smell of *M*. *foetida*, has been among the reasons provided as to why some individuals do not consume underutilised vegetables.

**Figure 5:**
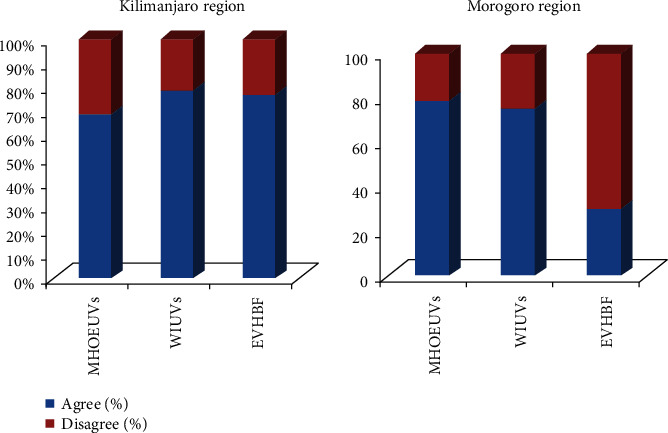
Interests and preferences of the UVs. MHOEUVs: members of the household often eat UVs; WIUVs: we are interested with the UVs; EVHBF: exotic vegetables have better features/benefits.

It was found that over 70% of the respondents often eat UVs in their households, and over 75% were interested in their consumption (Figure 5), implying that majority of the people in the study areas were interested in and utilised the UVs. However, according to various reports, the decline in their production and consumption in many rural communities in Africa is believed to be due to the introduction of exotic vegetable varieties [28, 29].

Among the reasons pointed out by the respondents as to why they used the UVs include (i) cutting short budget expenditure on exotic vegetable species due to their affordability; (ii) they grow organically and do not involve the use of inorganic fertilizers; (iii) they are potential not only as food but also as medicine; (iv) they are appetizers as their taste is good compared to the exotic species; (v) they can be used as accompaniments with any kind of food, including cooked rice, bananas, and stiff porridge (ugali).

### 3.3. Lifestyle/Image of Consumers concerning UVs

Respondents (70%) who considered the UVs as part of their diets had a positive image on them. They did not consider them as a poor man's diet or a primitive foodstuff. Instead, they had a strong preference for the vegetables such that some pointed out that they could not afford a meal without such vegetables. Some went to the extent of mocking people in the current generation that they were weak and sickling just because they consumed foods that did not add anything to boost their immunity system.

**Figure 6:**
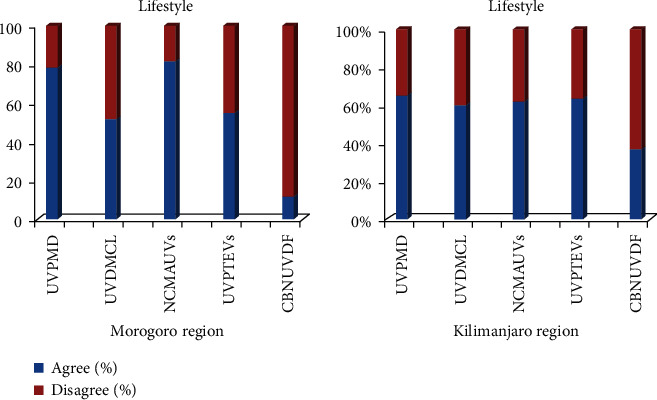
Lifestyle/image of consumers concerning UVs. UVPMD: UVs are considered as a “poor man's diet”; UVDMCL: UVs do not match consumers' lifestyle/image; NCMAUVs: nonconsumers have misconceptions about UVs; UVPTEVs: UVs are preferred to exotic vegetables; CBNUVDF: consumers have a basic need that UVs do not fulfill.

It was claimed that the UVs were pure and safe, because they did not involve any organic fertilisers or pesticides during their growing period. Nevertheless, in most cases, the rest of the respondents (20%) lacked interest in UVs and considered them as out of fashion and associated them with low-class people, also as food and source of income for the poor and unemployed households [30]. Further reports show that these misconceptions still linger in some places in Africa and will take time to change. Few of the respondents (25%) claimed that UVs were considered as poor man's diet, and those who consumed them were primitive and old-fashioned. Most of the midaged people have also fallen into that trap of misconceptions, where many of whom are the educated and people from the so called "well-off families." They (20%) are claiming that underutilised vegetables do not match their lifestyles or image (Figure 6).

### 3.4. Marketability

**Figure 7:**
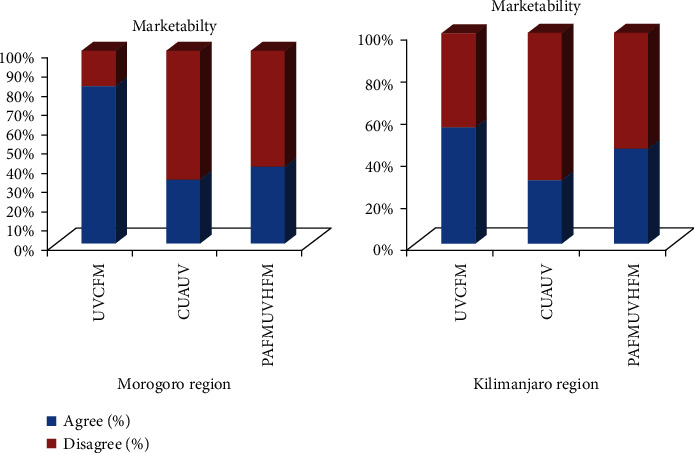
Marketability of the UVs. UVCFM: underutilised vegetables can be found in the market; CUAUV: consumers are unaware of the availability of underutilised vegetables; PAFMUVHFM: poor availability in the farms makes UVs hard to find in the market.

Over 70% of the respondents from both regions were aware of the availability of underutilised vegetables that they could be found even in the markets (Figure 7). Thus, low availability in the farms was shown by the natives to be an illogical reason for unavailability of UVs in the market (Figure 7). In Morogoro region, the markets for UVs were Morogoro town market, Mawenzi market, and Kimamba and Kilosa markets. For Kilimanjaro region, UVs were sold in Kalali, Mula, Kibororoni, Manyeni, and Mbuyuni. Also, other common marketing areas were church auctions and from street vendors. The density of the target market was small and poor. Those who bought the vegetables were people who regularly consumed the vegetables and those who had at least some knowledge concerning them.

UVs are highly perishable with a very short shelf life, deteriorating very quickly in terms of quality and flavour after harvesting, thus making the extent of postharvest losses huge if the crop was handled poorly. The ability to maintain freshness of the UVs obtained from the farms was a challenging task, and it was reported to be one of the major setbacks of farmers who would wish to expand their marketing opportunities and ability to compete in the marketplace. According to Schippers [31], this creates problems in the marketing chain with producers, traders, or consumers.

### 3.5. Affordability of the UVs

**Figure 8:**
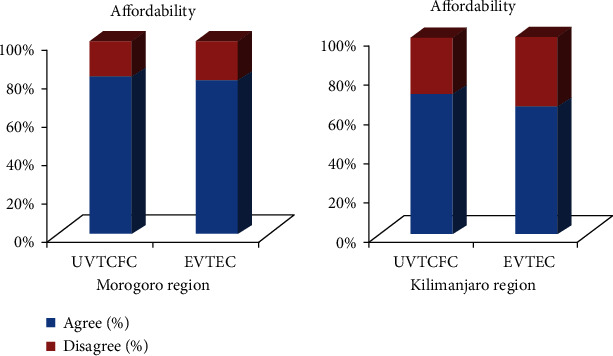
Affordability of the UVs. UVTCFC; underutilised vegetables are too cheap for consumers; EVTEC; exotic vegetables are too expensive for some consumers.

Over 75% of the respondents in the study agreed that the UVs were cheap and very affordable compared to the exotic vegetable species, which were too expensive (Figure 8). This was in line with Shumsky et al. [4] who reported that in poorer rural households, due to the fact that UVs cost less, the limited cash resources can instead be relocated to energy, shelter, food, and medical needs. This is further supported by Prajapati et al. [25], who reported that UVs can be gathered without monetary cost and do not require expensive inputs, machinery, or processing.

## 4. Conclusion

There are many varieties of UVs in Morogoro and Kilimanjaro regions. However, the UVs are currently showing decreasing usage due to the fads and myths surrounding them and the introduction of exotic species of vegetables, mainly due to the difficulties in preparation, processing techniques, palatability, negative image upon consumers, and their scarcity in the urban areas.

## 5. Recommendations

There is an urgent need for sensitizing and encouraging the local communities to increase consumption of these UVs to counter and change their negative image and attitudes towards these vegetables. Ways need to be developed to acquire seeds of the selected UVs for domestication so as to ensure a wider adoption on a sustainable basis.

## Figures and Tables

**Table 1:** List of underutilised vegetables reported by respondents in the study areas.

Local name	Scientific name	Edible portion	Data source (ward)
Sunga/Chunga/Mchunga	*Launea cornuta*	Leaf	Morogoro
Kikunde mbala	*Vigna vexillata*	Leaf	Morogoro
Mlenda mgunda	*Corchorus olitorius*	Leaf; stem	Morogoro
Mbwimbwiza	Unidentified	Leaf	Morogoro
Mchicha pori	*Amaranthus* spp.	Leaf; stem	Morogoro
Songolomaridadi	Unidentified	Leaf	Morogoro
Mnafu pori (bwasi)	*Solanum incanum*	Leaf	Morogoro
Kigegedu/Chamgegedu/Chainizi pori	Unidentified	Leaf	Morogoro
Kidingililu	Unidentified	Flower	Morogoro
Mhilile	Unidentified	Leaf	Morogoro
Mlenda mwage	*Sesbania* spp.	Leaf	Morogoro
Mgange	Unidentified	Leaf	Morogoro
Mangwi	Unidentified	Leaf	Morogoro
Mlenda mwidu	*Corchorus* spp.	Leaf	Morogoro
Mwanamdewa	Unidentified	Leaf	Morogoro
Matembele pori	*Ipomeas* spp.	Leaf	Morogoro
Nyakatwanga	Unidentified	Leaf	Morogoro
Kikongwa	Unidentified	Leaf	Morogoro
Orobwe	Unidentified	Leaf	Morogoro
Msimwe	Unidentified	Leaf	Morogoro
Koroga	Unidentified	Leaf	Morogoro
Esikisilanjoi	Unidentified	Leaf	Morogoro
Nyaweza	Unidentified	Leaf	Morogoro
Mokiki	*Momordica foetida*	Leaf	Kilimanjaro
Ibangasa	Unidentified	Leaf	Kilimanjaro
Ngolowo	Wild cowpea	Leaf	Kilimanjaro
Kisamvu	*Manihot grazioli*	Leaf	Kilimanjaro
Makamaka/Mbaro	*Bidens pilosa*	Leaf	Kilimanjaro
Majani ya kunde	*Vigna* spp.	Leaf	Kilimanjaro
Mnafu	*Solanu nigrum*	Leaf	Kilimanjaro
Mashona nguo	*Bidens pilosa*	Leaf	Kilimanjaro
Sunga/Kiruarua	*Launea cornuta*	Leaf	Kilimanjaro
Mchicha pori	*Amaranthus* spp.	Leaf	Kilimanjaro
Nyanya pori (Nukuria)	*Solanum betaceum*	Leaf	Kilimanjaro
Mgagani	*Cleome gynandra*	Leaf	Kilimanjaro
Masuti (Majani ya maboga)	*Cucurbita maxima*	Leaf	Kilimanjaro
Tungusha/tree tomato	*Solanum betaceum*	Leaf/fruit	Kilimanjaro
Mtasi	Unidentified	Leaf	Kilimanjaro
Inyiri	*Basella alba*	Leaf	Kilimanjaro
Kimashira	Unidentified	Leaf	Kilimanjaro
Mchunga,	*Launea cornuta*	Leaf	Kilimanjaro
Mashona nguo (Chagga: Makamaka)	*Bidens pilosa*	Leaf	Kilimanjaro
Mlenda pori	*Corchorus* spp.	Leaf	Kilimanjaro
Kunde pori	*Vigna vexillata*	Leaf	Kilimanjaro

**Table 2:** List of the selected underutilised vegetables from the study areas.

Local name	English name	Swahili name	Scientific name
Sunga	Bitter lettuce	Mchunga	*Launea cornuta*
Kikundembala	Wild cowpea	Kunde pori	*Vigna vexillata*
Mokiki	Bitter cucumber	—	*Momordica foetida*
Inyiri	Malabar spinach	Delega	*Basella alba*

## Data Availability

The datasets used and/or analysed during the current study are available from the corresponding author on reasonable request.
